# Supplementary material for: YTHDF1 links hypoxia adaptation and non-small cell lung cancer progression
Source: Nat Commun. 2019 Oct 25;10:4892. doi: 10.1038/s41467-019-12801-6 (PMC6814821; doi:10.1038/s41467-019-12801-6)
Supplement: Supplementary file 1 — Supplementary Information [file 41467_2019_12801_MOESM1_ESM.pdf]

Supplementary Information

YTHDF1 links hypoxia adaptation and non-small cell lung cancer progression

Shi et al.

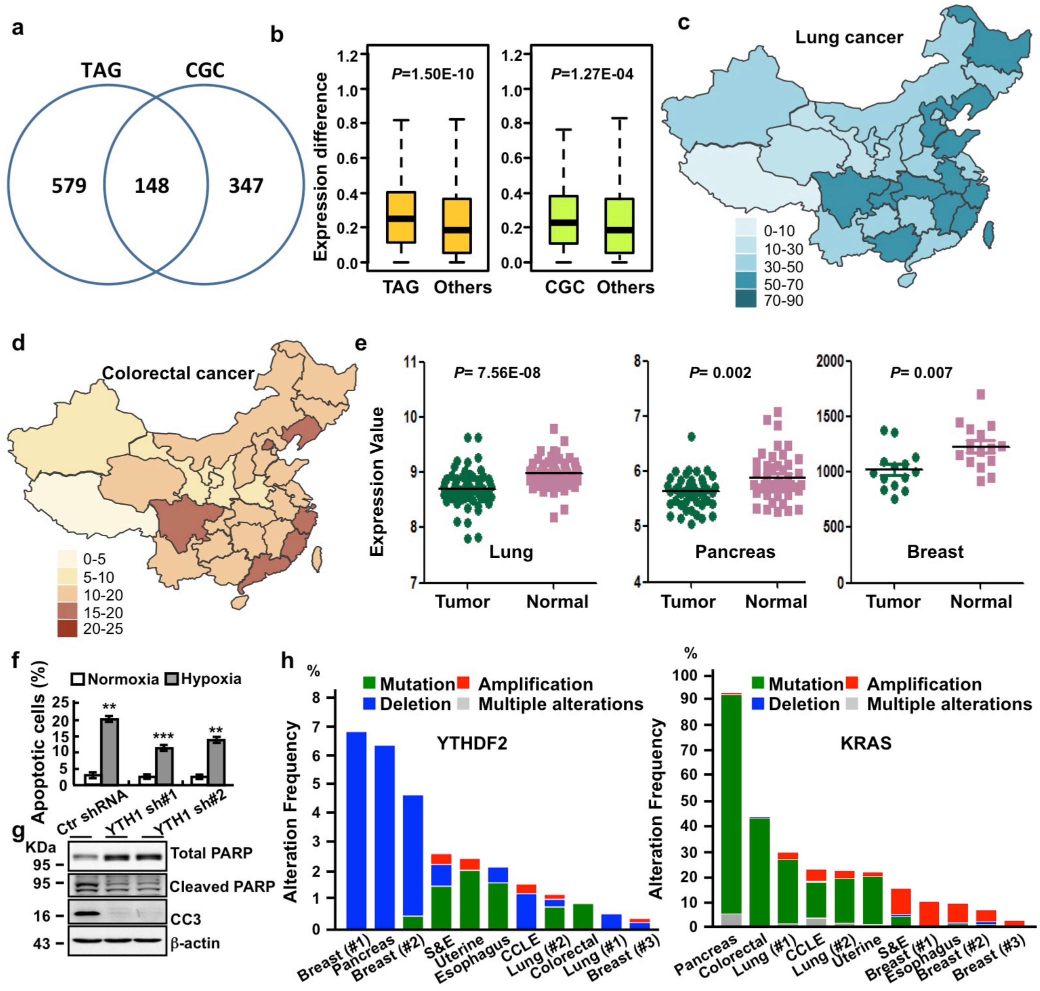

Supplementary Fig. 1: Identification of *TEX2* and *YTHDF1*. **a,** The common and different genes between TAG and CGC. **b,** There is a higher level of expression difference for TAG and CGC than that for other genes. Expression difference for each gene was calculated by absolute difference of log2 (FPKM+1) values of gene in Tibetan pig lung and Min pig lung. **c-d,** Age-standardised rates of death (per 100,000 people) for lung (c) and colorectal (d) cancers in different provinces for men. The data were downloaded from the study [27]. Details for calculating age-standardised rates of death can be found in the original paper. Data for Taiwan is not available. **e,** Significant differential expression of *TEX2* between tumor and normal tissues from lung (GEO accession code: GSE19804), pancreas (GSE28735) and breast (GSE9574) cancers. **f,** Quantification data for Fig. 1h. **g,** Western blot showed that deficiency of YTHDF1 reduced cleave caspase-3 (CC3) and PARP expression upon 1% O_2 treatment. **h,** The mutation patterns for YTHDF2 and KRAS in various cancers. Mutation (green), deletion (blue), amplification (red), multiple alterations (grey).

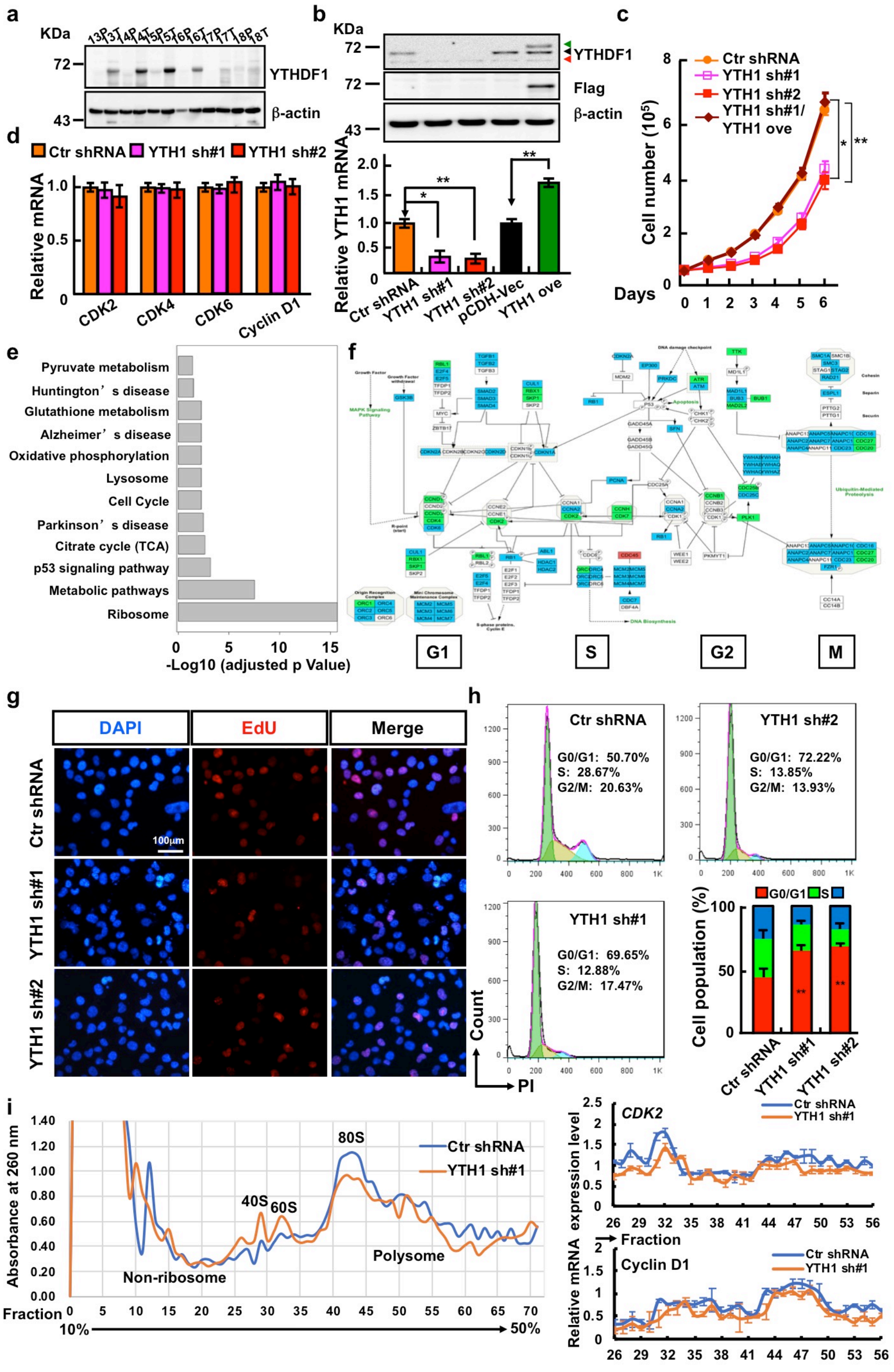

Supplementary Fig. 2: YTHDF1 inhibits cell proliferation. **a,** YTHDF1 is increased in NSCLC cancerous tissues examined by western blot. Additional samples were tested. **b,** Establishment of YTHDF1 overexpression and knockdown H1299 cell lines, verified by western blot (top) and Real-time RT-PCR (bottom). Green arrow: exogenous YTHDF1-Flag; black arrow: Endogenous YTHDF1; red arrow: non-specific band. Antibodies: YTHDF1, Flag and β-actin. **c,** Knockdown of YTHDF1 dramatically inhibits H1299 cell proliferation in vitro. **d,** YTHDF1 does not regulate the mRNA expressions of CDK2/4/6 and cyclin D1. **e,** 1363 significantly changed proteins were mapped

to the KEGG (Kyoto Encyclopedia of Genes and Genomes) pathway database, and 12 pathways were enriched with p value <0.05. **f,** Indicated proteins were mapped to the cell cycle. Red: up-regulated; blue: no change (or change less than 1.2-fold); green: down-regulated. **g,** Effect of YTHDF1 knockdown on DNA synthesis in H1299 cells, as measured with a Click-iT EdU Alexa Fluor Imaging Kit. Scale bar: 100μM. **h,** Effect of YTHDF1 knockdown on the G0/G1 cell population in H1299 cells, as measured by PI staining and flow cytometry. Quantification data was also included. **i,** Polysomal profiling of endogenous CDK2 and cyclin D1 mRNAs in YTHDF1 knockdown or scramble shRNA control A549 cells. n=3 independent experiments. Means±SEM, * P <0.05; ** P <0.01; t-test.

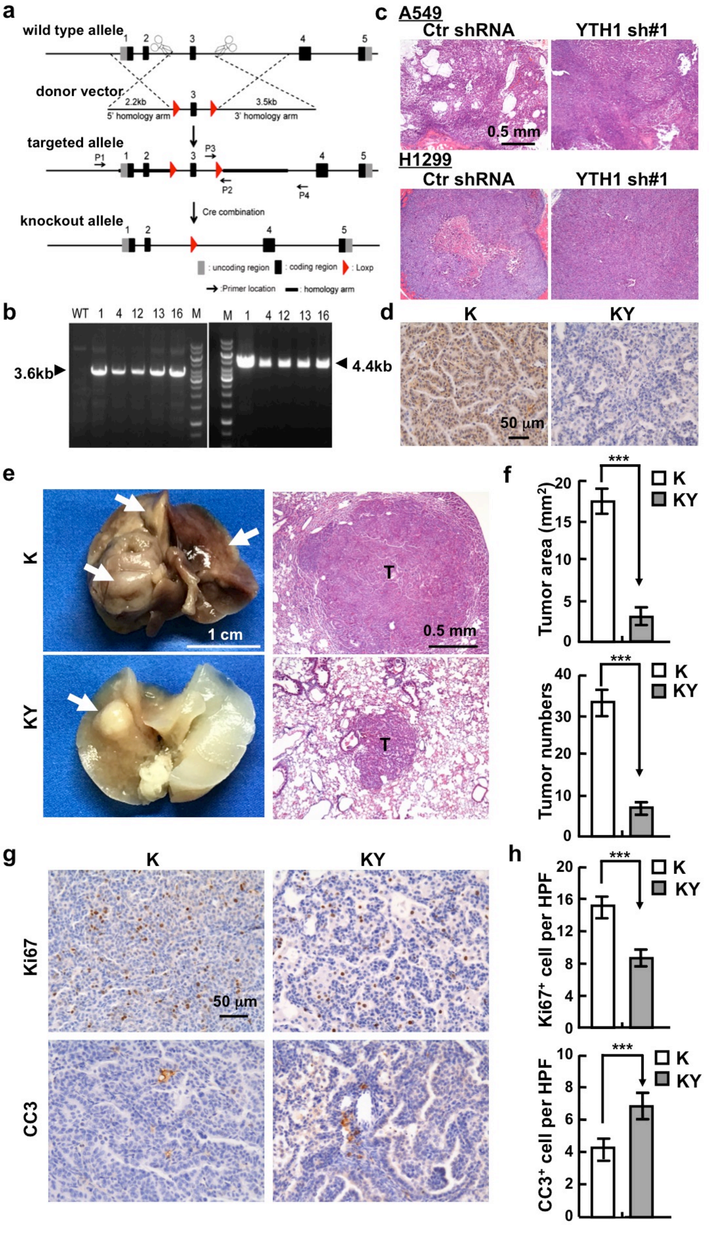

Supplementary Fig. 3: YTHDF1 depletion inhibits *de novo* ADC progression. **a,** Schematic representation of the recombination strategy for *YTHDF1*$^{lox/lox}$ mouse. **b,** PCR identification of *YTHDF1*$^{lox/lox}$ positive F1 generation mice using primers P1-P4 (primers information were indicated in Materials and Methods). Primer sets P1 and P2, were used to identify the recombination of 5'-homology arm, which yielded one fragment (3.6 kb) in positive mice (left); primer sets P3 and P4, were used to identify the recombination of 3'-homology arm, which yielded one fragment (4.4 kb) in positive mice (right). (WT: wild type mouse genomic DNA control; 1,4,12,13,16: positive F1 generation mice; M: Fermentas 1kb DNA ladder). **c,** Representative images of H&E-stained lung sections of indicated xenograft tumors. Scale bar: 0.5 mm. **d,** YTHDF1 is upregulated in ADC from K mice compared with the complete loss of YTHDF1 from KY mouse tumors. **e-f,** Representative images of H&E-stained lung sections of K or KY mice at 22 weeks after viral administration (e). Quantification data for the tumor numbers and tumor size ($\geqslant 1mm^2$) in K or KY mice (f). Scale bars are indicated in each image. T: Tumor tissues. **g-h,** Representative images of Ki67 and CC3 immunostaining (g) of lung tumors and statistical analyses of the Ki67- and CC3-positive index in the indicated genotype (h), respectively. Scale bar, 50 μm. Means $\pm$ SEM, * P <0.05; ** P <0.01; *** P <0.001; t-test.

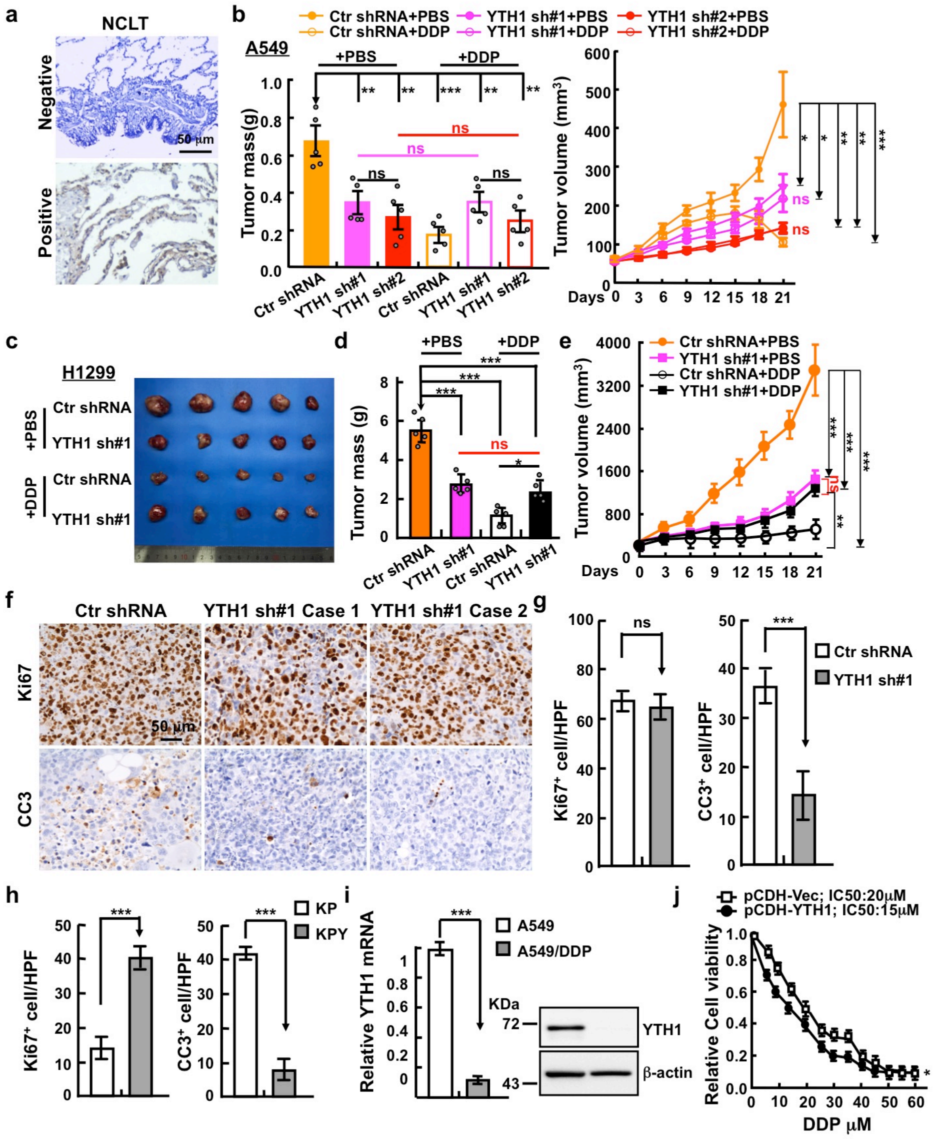

Supplementary Fig. 4: YTHDF1 knockdown promotes DDP resistance. **a,** The negative and positive expression patterns of YTHDF1 protein were respectively shown in the non-cancerous control lung tissue (NCLT). **b,** Effect of YTHDF1 depletion on A549 xenograft tumor weight and volumes in male nude mice in PBS groups. YTHDF1 knockdown tumors showed resistance to DDP treatment compared to control shRNA tumors. **c,** Xenograft tumor masses harvested from indicated mice treated by indicated conditions. DDP: 7mg/kg, once a week for 3 weeks. **d-e,** YTHDF1 elimination inhibits H1299 xenograft tumor weight (d) and volumes (e) in male nude mice in PBS groups, and YTHDF1 knockdown tumors showed resistance to DDP treatment compared to control shRNA tumors. **f-g,** Representative IHC staining for indicated xenograft tumors after DDP treatment (f). Scale bar: 50μm. Antibodies are: Ki67 and CC3. (g) is the quantification data for (f). **h,** Quantification data for Ki67 and CC3 in Fig. 4f. **I,** YTHDF1 is decreased in A549/DDP cells compared to that in A549, validated by both Real-time RT-PCR (left) and western blot (right). **j,** Transient forced

expression of YTHDF1 sensitizes A549/DDP cells responding to DDP treatment. Means±SEM, * P <0.05; ** P <0.01; *** P <0.001; t-test. P=ns: no significant difference.

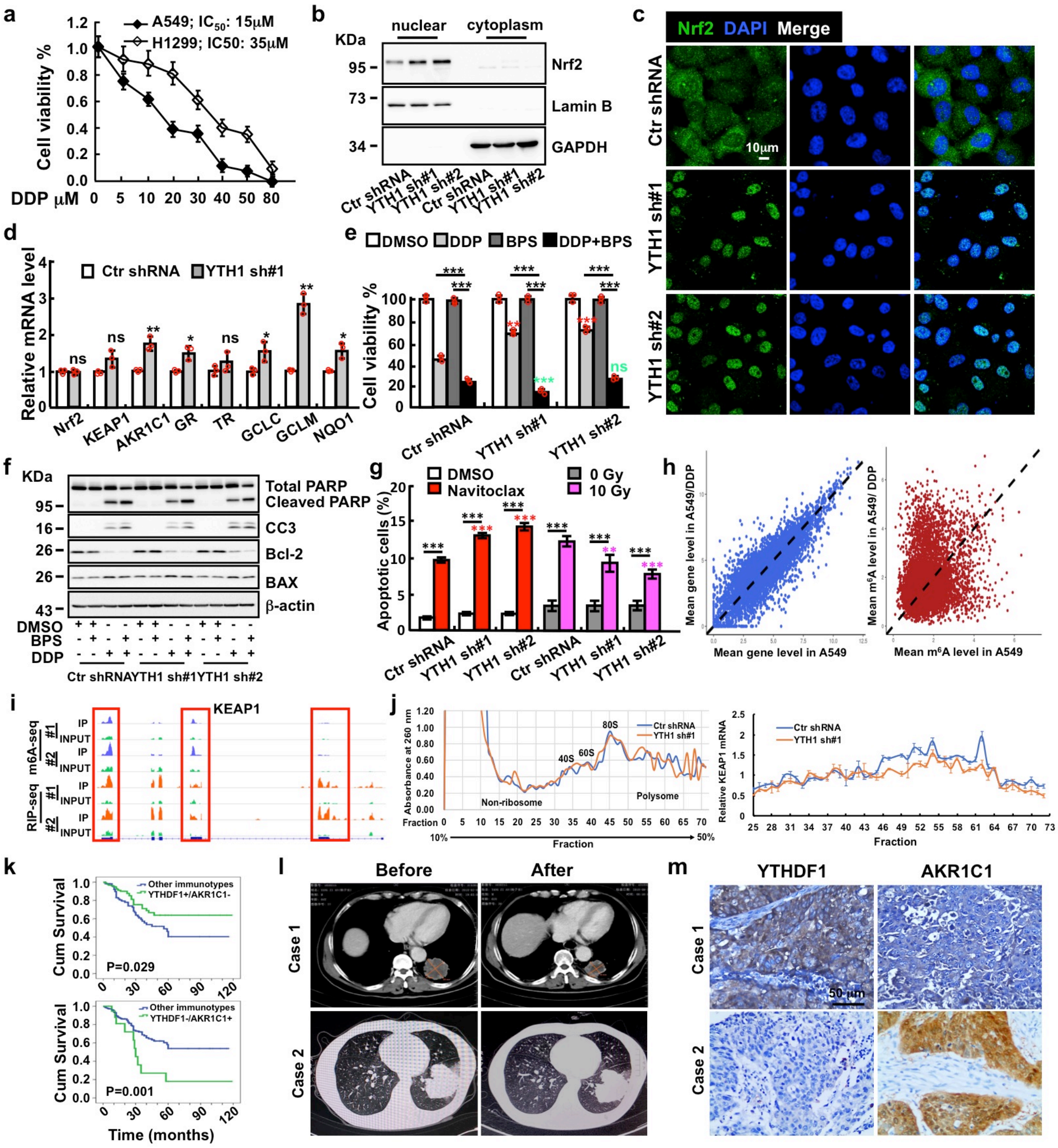

Supplementary Fig. 5: YTHDF1 functions through Keap1-Nrf2-AKR1C1 axis. **a,** IC_{50} verification for DDP in A549 and H1299. **b,** After DDP (35μM) treatment, indicated cell extracts from H1299 were examined by western blot. Antibodies: Nrf2, Lamin B (Nuclear fraction), GAPDH (cytosol fraction). **c,** Representative images indicating Nrf2 nuclear accumulation after YTHDF1 knockdown in H1299 cells treated by 35μM DDP. Scale bar: 10μm. **d,** Relative mRNA expression levels of indicated genes in A549 cells after DDP treatment by RT-PCR. **e-f,** Effect of DDP and/or AKR1C1 inhibitor BPS on cell viabilities of indicated H1299 cell lines (e), which were further validated by western blot (f). DDP: 35μM; BPS: 40μM. Indicated cells were pretreated by DDP for 12 h followed by BPS treatment.

Indicated total extracts were probed with indicated antibodies: PARP, CC3, Bcl-2, BAX and β-actin. Red stars: DDP treatment group comparison; Green stars: DDP+BPS treatment group comparison. **g,** Blank and Red columns: indicated cells were treated with DMSO or Navitoclax (15μM); grey and pink columns: indicated cells were treated with 0 Gy and 10 Gy radiation. Cells were collected and stained with Annexin V/PI, and the percentage of apoptotic cells was assessed by flow cytometry. Red stars: navitoclax treatment group comparison; pink stars: radiation treatment group comparison. **h,** The gene expression and m^6A levels in A549 and A549/DDP cells were compared. **i,** IGV tracks displaying m^6A peaks and YTHDF1 binding enrichment in Keap1 mRNA from m^6A-seq and YTHDF1 RIP-seq in A549 cells, respectively. Blue indicated m^6A-seq IP, orange indicated YTHDF1 RIP-seq IP, and green indicated INPUT. **j,** Polysomal profiling of endogenous Keap1 mRNAs in YTHDF1 knockdown or scramble shRNA control A549 cells treated by DDP (15μM). n=2 independent experiments. **k,** The overall survival rates were validated for indicated immunotypes. **l,** Representative computerized tomography (CT) scan images of NSCLC responders (case 1) and non-responders (case 2) before and after platinum based neoadjuvant chemotherapy. **m,** Representative images of YTHDF1 and AKR1C1 IHC staining in NSCLC responders (case 1) and non-responders (case 2) tumor tissues to platinum based neoadjuvant chemotherapy. Means±SEM, * P <0.05; ** P <0.01; *** P <0.001; t-test.

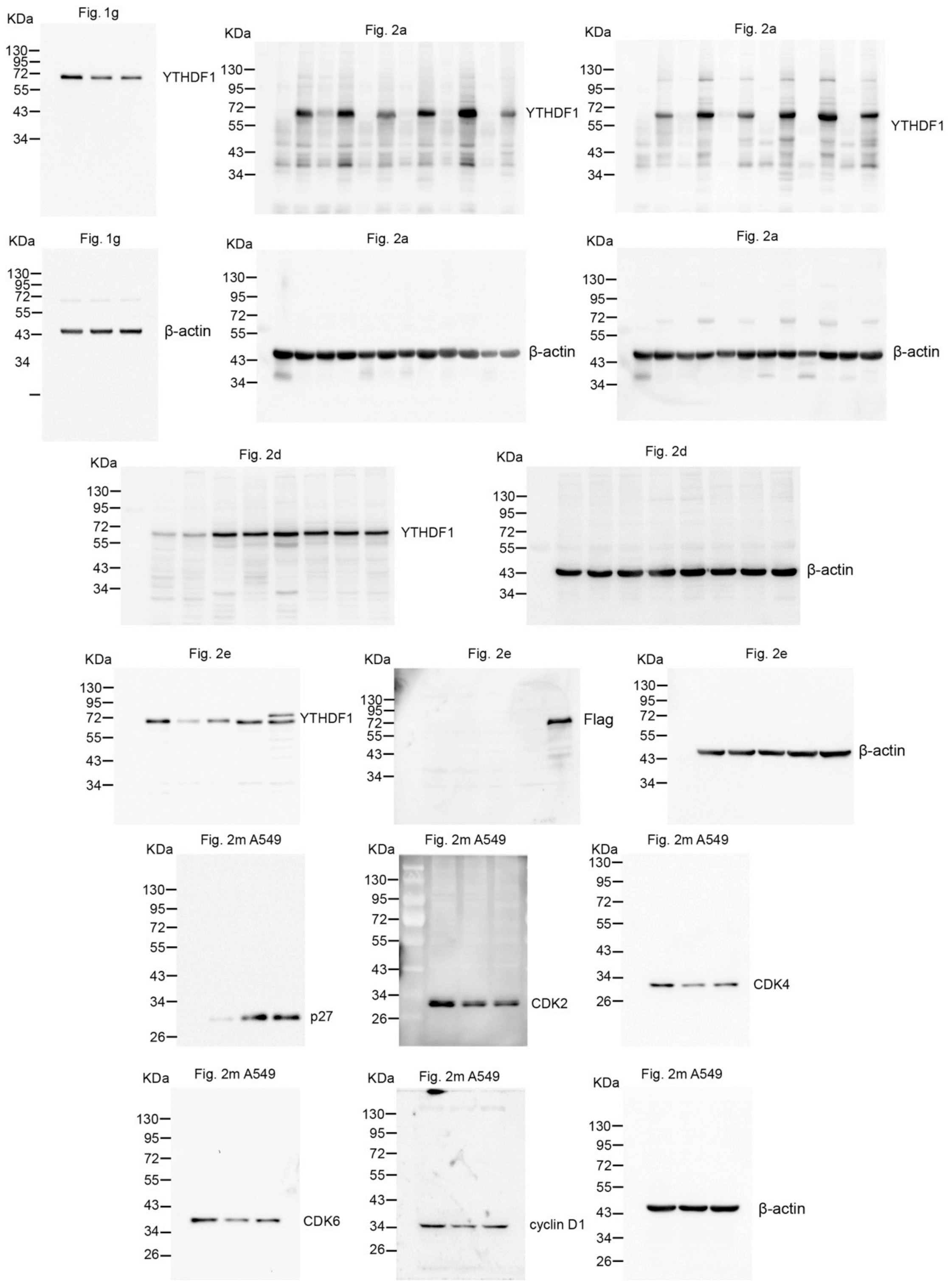

Fig. 1g
KDa
130
95
72
55
43
34
YTHDF1
Fig. 2a
KDa
130
95
72
55
43
34
YTHDF1
Fig. 2a
KDa
130
95
72
55
43
34
YTHDF1
Fig. 1g
KDa
130
95
72
55
43
34
β-actin
Fig. 2a
KDa
130
95
72
55
43
34
β-actin
Fig. 2a
KDa
130
95
72
55
43
34
β-actin
Fig. 2d
KDa
130
95
72
55
43
34
YTHDF1
Fig. 2d
KDa
130
95
72
55
43
34
β-actin
Fig. 2e
KDa
130
95
72
55
43
34
YTHDF1
Fig. 2e
KDa
130
95
72
55
43
34
Flag
Fig. 2e
KDa
130
95
72
55
43
34
β-actin
Fig. 2m A549
KDa
130
95
72
55
43
34
26
p27
Fig. 2m A549
KDa
130
95
72
55
43
34
26
CDK2
Fig. 2m A549
KDa
130
95
72
55
43
34
26
CDK4
Fig. 2m A549
KDa
130
95
72
55
43
34
26
CDK6
Fig. 2m A549
KDa
130
95
72
55
43
34
26
cyclin D1
Fig. 2m A549
KDa
130
95
72
55
43
34
26
β-actin

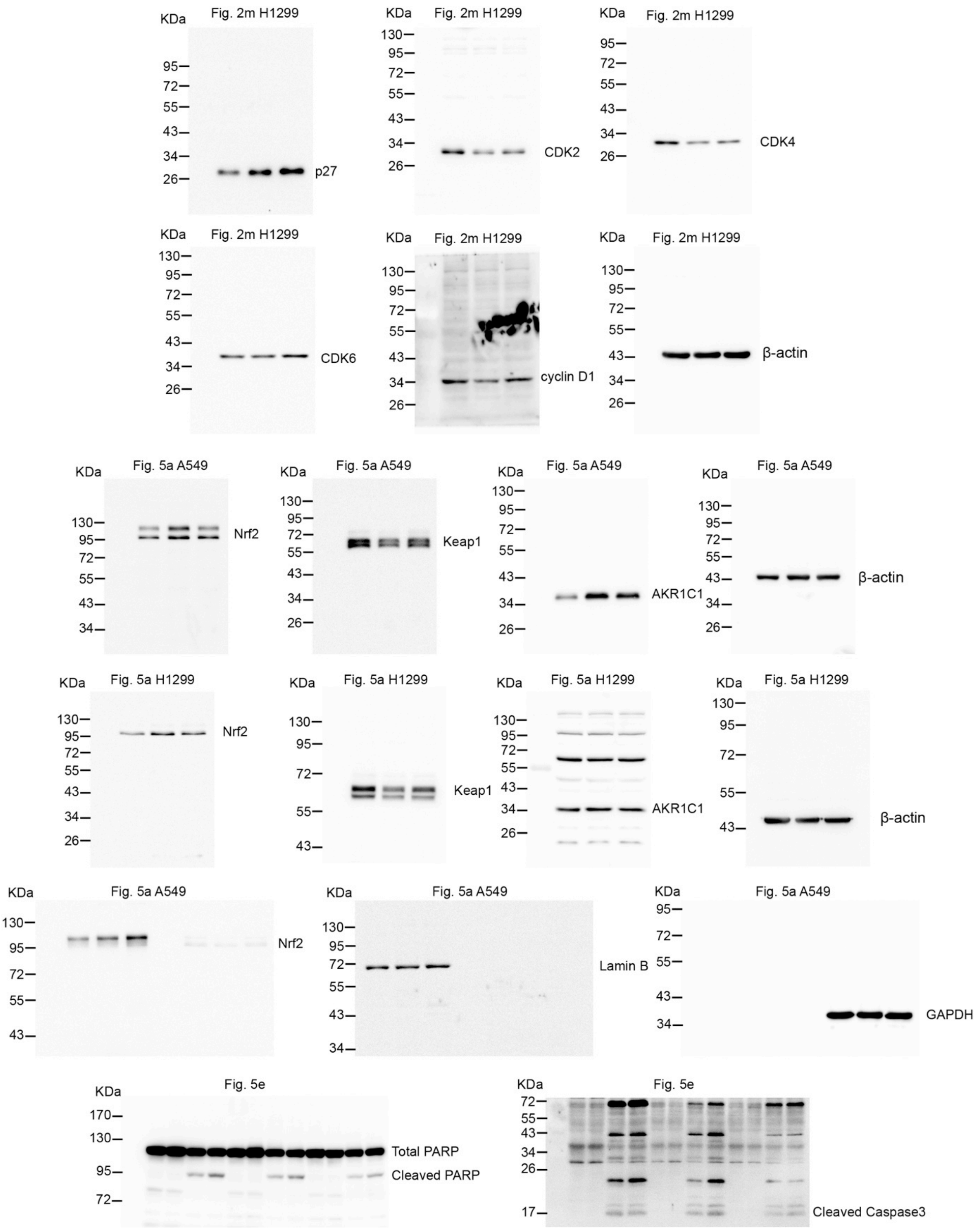

KDa
Fig. 2m H1299
95
72
55
43
34
26
p27
KDa
Fig. 2m H1299
130
95
72
55
43
34
26
CDK2
KDa
Fig. 2m H1299
95
72
55
43
34
26
CDK4
KDa
Fig. 2m H1299
130
95
72
55
43
34
26
CDK6
KDa
Fig. 2m H1299
130
95
72
55
43
34
26
cyclin D1
KDa
Fig. 2m H1299
130
95
72
55
43
34
26
β-actin
KDa
Fig. 5a A549
130
95
72
55
43
34
Nrf2
KDa
Fig. 5a A549
130
95
72
55
43
34
26
Keap1
KDa
Fig. 5a A549
130
95
72
55
43
34
26
AKR1C1
KDa
Fig. 5a A549
130
95
72
55
43
34
26
β-actin
KDa
Fig. 5a H1299
130
95
72
55
43
34
26
Nrf2
KDa
Fig. 5a H1299
130
95
72
55
43
Keap1
KDa
Fig. 5a H1299
130
95
72
55
43
34
26
AKR1C1
KDa
Fig. 5a H1299
130
95
72
55
43
β-actin
KDa
Fig. 5a A549
130
95
72
55
43
Nrf2
KDa
Fig. 5a A549
130
95
72
55
43
34
Lamin B
KDa
Fig. 5a A549
95
72
55
43
34
GAPDH
KDa
Fig. 5e
170
130
95
72
Total PARP
Cleaved PARP
KDa
Fig. 5e
72
55
43
34
26
17
Cleaved Caspase3

Fig. 5e

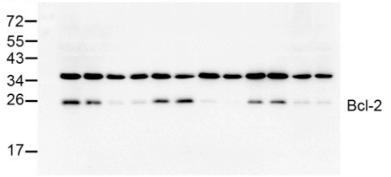

Fig. 5e

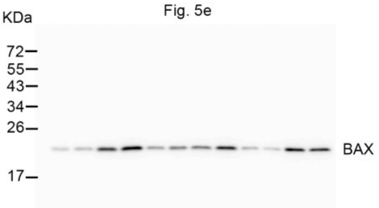

Fig. 5e

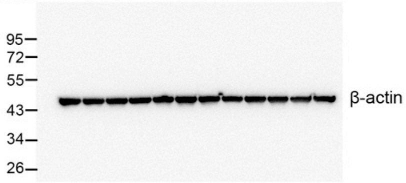

Fig. 5h

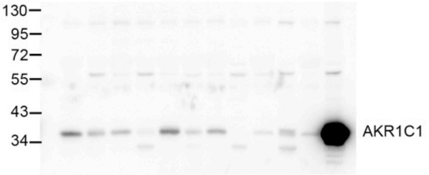

Fig. 5h

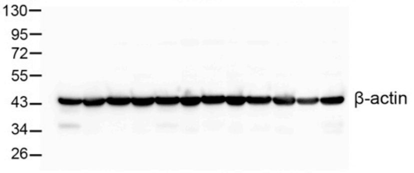

Fig. 5h

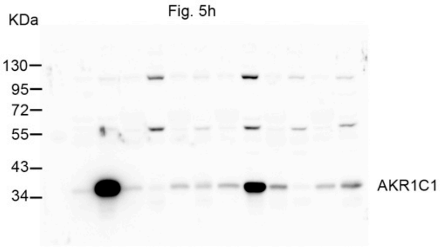

Fig. 5h

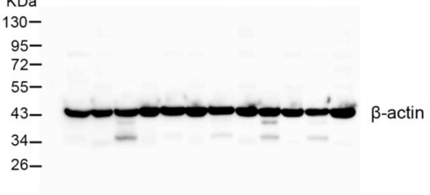

Fig. 5h

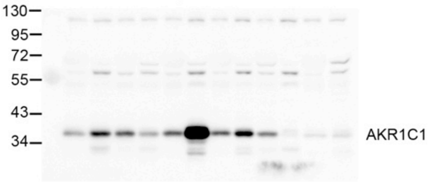

Fig. 5h

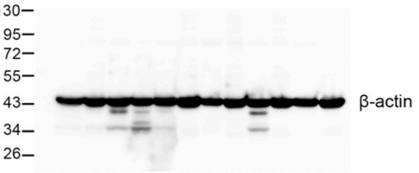

Supplementary Fig. 1g

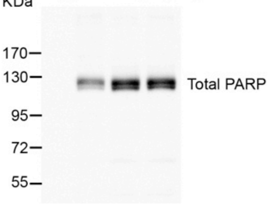

Supplementary Fig. 1g

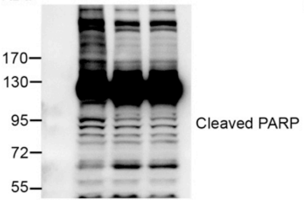

Supplementary Fig. 1g

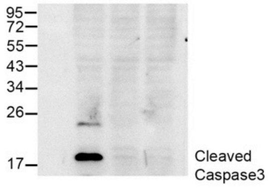

Supplementary Fig. 1g

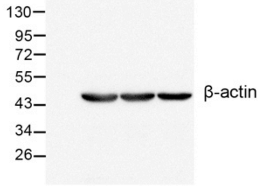

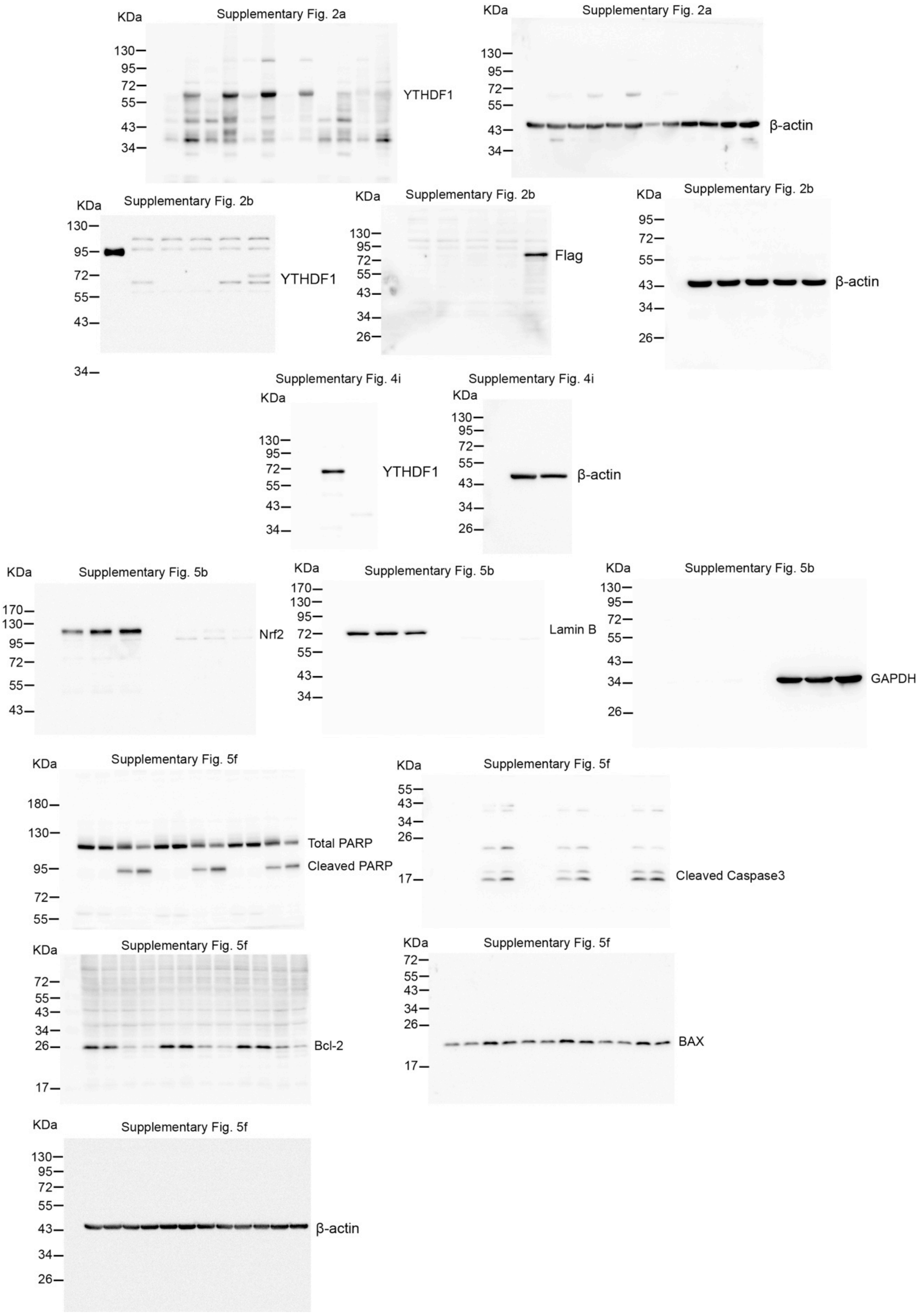

Supplementary Fig. 6 Uncropped scans of western blots

Supplementary Table 1. The cBioPartal database query used in Fig. 1j and Supplementary Fig. 1h.

Cancer type	DOI	Journal
Breast (#1)	doi: 10.1038/nature13952	Nature
Pancreas	doi: 10.1038/ncomms7744	Nature communications
Breast (#2)	doi: 10.1371/journal.pmed.1002201	PLoS medicine
S&E	doi: 10.1038/nature20805	Nature
CCLE	doi: 10.1038/nature11003	Nature
Esophagus	TCGA	
Breast (#3)	doi: 10.1038/ncomms11479	Nature communications
Colorectal	TCGA	
Lung (#1)	doi: 10.1016/j.cell.2012.08.029	Cell
Uterine	TCGA	
Lung (#2)	doi: 10.1038/ng.3564	Nature genetics

Supplementary Table 2. Clinicopathological characteristics of patients with NSCLC and non-cancerous control lung tissues in the tissue arrays.

Patients characteristics	No. of patients (%)
**NSCLC**	
**Age(years)**	
≤50	192(39.4)
＞50	295(60.6)
**Gender**	
Male	366(75.2)
Female	121(24.8)
**Clinical stages**	
Stage Ⅰ	125(25.7)
Stage Ⅱ	128(26.3)
Stage Ⅲ	204(41.9)
Stage Ⅳ	30(6.1)
**Lymph node status**	
N0	207(42.5)
N1/N2/N3	280(57.5)
**Histological type**	
SCC	238(48.9)
ADC	249(51.1)
**Differentiation**	
Well	34(7.0)
Moderate	218(44.8)
Poor	235 (48.2)
**Non-cancerous lung tissues**	
**Age(years)**	
≤50	51(33.6)
＞50	101(66.4)
**Gender**	
Male	104(68.4)
Female	48(31.6)
