# Supplementary material for: YTHDF1 links hypoxia adaptation and non-small cell lung cancer progression
Source: Nat Commun. 2019 Oct 25;10:4892. doi: 10.1038/s41467-019-12801-6 (PMC6814821; doi:10.1038/s41467-019-12801-6)
Supplement: Supplementary file 3 — Description of Additional Supplementary Files [file 41467_2019_12801_MOESM3_ESM.pdf]

Description of Additional Supplementary Files

File Name: Supplementary Data 1
Description: Tibetan pigs and lowland Min pigs were examined by RNA-sequencing, and it was found that differentially expressed genes were significantly enriched in categories associated with cancer, cell death and apoptotic processes.

File Name: Supplementary Data 2
Description: Top two ranked positively selected genes from individual domestic animals and their functional roles involved in differential cancers.

File Name: Supplementary Data 3
Description: Proteomic analysis in YTHDF1 knockdown cells.

File Name: Supplementary Data 4
Description: m6A-seq and RIP-seq data

File Name: Supplementary Data 5
Description: Comparison of the m6A level in A549 and A549/DDP cells.

File Name: Supplementary Data 6
Description: Primer sequences and antibodies used in this study.
