# Supplementary material for: YTHDF1 links hypoxia adaptation and non-small cell lung cancer progression
Source: Nat Commun. 2019 Oct 25;10:4892. doi: 10.1038/s41467-019-12801-6 (PMC6814821; doi:10.1038/s41467-019-12801-6)
Supplement: Supplementary file 10 — Reporting Summary [file 41467_2019_12801_MOESM10_ESM.pdf]

nature research

Corresponding author(s): NCOMMS-18-32925B

Last updated by author(s): Aug 22, 2019

Reporting Summary

Nature Research wishes to improve the reproducibility of the work that we publish. This form provides structure for consistency and transparency in reporting. For further information on Nature Research policies, see Authors & Referees and the Editorial Policy Checklist.

Statistics

For all statistical analyses, confirm that the following items are present in the figure legend, table legend, main text, or Methods section.

n/a	Confirmed	
☐	☒	The exact sample size (*n*) for each experimental group/condition, given as a discrete number and unit of measurement
☐	☒	A statement on whether measurements were taken from distinct samples or whether the same sample was measured repeatedly
☐	☒	The statistical test(s) used AND whether they are one- or two-sided<br>*Only common tests should be described solely by name; describe more complex techniques in the Methods section.*
☒	☐	A description of all covariates tested
☒	☐	A description of any assumptions or corrections, such as tests of normality and adjustment for multiple comparisons
☐	☒	A full description of the statistical parameters including central tendency (e.g. means) or other basic estimates (e.g. regression coefficient) AND variation (e.g. standard deviation) or associated estimates of uncertainty (e.g. confidence intervals)
☒	☐	For null hypothesis testing, the test statistic (e.g. *F*, *t*, *r*) with confidence intervals, effect sizes, degrees of freedom and *P* value noted<br>*Give P values as exact values whenever suitable.*
☒	☐	For Bayesian analysis, information on the choice of priors and Markov chain Monte Carlo settings
☒	☐	For hierarchical and complex designs, identification of the appropriate level for tests and full reporting of outcomes
☒	☐	Estimates of effect sizes (e.g. Cohen's *d*, Pearson's *r*), indicating how they were calculated

Our web collection on statistics for biologists contains articles on many of the points above.

Software and code

Policy information about availability of computer code

Data collection	Expression data of genes YTHDF1 and TEX2 were downloaded from NCBI GEO with accession codes including GSE10072, GSE28735, GSE24514, GSE21422, GSE19804 and GSE9574.
Data analysis	The trimmed reads were aligned against the reference genome of Sus scrofa (Sscrfa 10.2) using TopHat v2.0.4 with default parameters and using the gene annotation available at Ensembl v77. The XP-EHH value for each SNP was calculated by the XP-EHH program (http://hgdp.uchicago.edu/Software/). ΔDAF (the difference of derived allele frequencies) was calculated for each SNP as the derived allele frequency (DAF) in the domestic animals in highland minus the DAF in domestic animals from the lowland. FST, XP-EHH, and ΔDAF (the difference of the derived allele frequencies) were integrated to identify potential positive selection in the Tibetan domestic mammals, where i is different methods and Ps represent the probability under positive selection. The iFXD value of each protein encoding gene was calculated by averaging the iFXD values of all SNPs within a gene.

For manuscripts utilizing custom algorithms or software that are central to the research but not yet described in published literature, software must be made available to editors/reviewers. We strongly encourage code deposition in a community repository (e.g. GitHub). See the Nature Research guidelines for submitting code & software for further information.

Data

Policy information about availability of data

All manuscripts must include a data availability statement. This statement should provide the following information, where applicable:

- Accession codes, unique identifiers, or web links for publicly available datasets
- A list of figures that have associated raw data
- A description of any restrictions on data availability

The datasets generated during and/or analysed during the current study are available from the corresponding author on reasonable request.

Field-specific reporting

Please select the one below that is the best fit for your research. If you are not sure, read the appropriate sections before making your selection.

☒ Life sciences ☐ Behavioural & social sciences ☐ Ecological, evolutionary & environmental sciences

For a reference copy of the document with all sections, see nature.com/documents/nr-reporting-summary-flat.pdf

Life sciences study design

All studies must disclose on these points even when the disclosure is negative.

Sample size	Sample sizes and number of replicates were chosen basing on that reproducibility can be established and statistical significance can be reached.
Data exclusions	In the quantitative western blot analyses, transformants not expressing the indicated protein were excluded. Also, bands of proteins which were apparently not blotted were excluded from the statistical analysis.
Replication	All experiments were repeated at least 3 times. * P <0.05; ** P <0.01; *** P <0.001; t-test.
Randomization	Five-week old male nude mice were randomly divided into indicated groups and injected with cell lines stably expressing scrambled control shRNA or shRNAs targeting YTHDF1.
Blinding	Investigators were blinded to group allocation during BrdU incorporation and Nrf2 staining data collection and/or analysis.

Reporting for specific materials, systems and methods

We require information from authors about some types of materials, experimental systems and methods used in many studies. Here, indicate whether each material, system or method listed is relevant to your study. If you are not sure if a list item applies to your research, read the appropriate section before selecting a response.

Materials & experimental systems

n/a	Involved in the study	
☐	☒	Antibodies
☐	☒	Eukaryotic cell lines
☒	☐	Palaeontology
☐	☒	Animals and other organisms
☐	☒	Human research participants
☐	☒	Clinical data

Methods

n/a	Involved in the study	
☒	☐	ChIP-seq
☐	☒	Flow cytometry
☒	☐	MRI-based neuroimaging

Antibodies

Antibodies used	Rabbit anti-YTHDF1 (#nbp1-52865, NOVUS); Rabbit anti-YTHDF1 (#17479-1-AP, Proteintech); Mouse anti-p27 (#610241, BD); Rabbit anti-CDK2 (#10122-1-AP, Proteintech); Rabbit anti-CDK4 (#ab108357,abcam); Rabbit anti-CDK6(#ab124821,abcam); Mouse anti-cyclin D1(#60186-1-1g,Proteintech); Rabbit anti-Laminb1(#ab16048,abcam); Mouse anti-β-actin (#60008-1-1g,Proteintech); Mouse anti-GAPDH (#60004-1-1g,Proteintech); Rabbit anti-PARP (#9542S, Cell Signaling Technology); Rabbit anti-Cleaved Caspase3 (#9661S, Cell Signaling Technology); Mouse anti-Bcl-2 (#15071S, Cell Signaling Technology); Mouse anti-BAX (#ab77566, abcam); Rabbit anti-Nrf2 (#ab62352, abcam); Rabbit anti-KEAP1 (#10503-2-AP, Proteintech); Rabbit anti-AKR1C1 (#ab192785, abcam)
Validation	Documents certify that the products have met the quality control standards defined by the manufacturers.

Eukaryotic cell lines

Policy information about cell lines

Cell line source(s)	Cell line sources are listed in Methods section.
Authentication	The lines were authenticated by short tandem repeat (STR) profiling.
Mycoplasma contamination	The cell lines were all tested negative for mycoplasma contamination.
Commonly misidentified lines (See ICLAC register)	No commonly misidentified cell lines were used in this study.

Animals and other organisms

Policy information about studies involving animals; ARRIVE guidelines recommended for reporting animal research

Laboratory animals	Species: C57BL/6, nude mice; sex: male; age: 4-6 weeks old.
Wild animals	This study did not involve wild animal.
Field-collected samples	The study did not involve samples collected from the field.
Ethics oversight	Mouse care and treatment was approved by the Animal Care and Use Committee at the Kunming Institute of Zoology, Chinese Academy of Sciences.

Note that full information on the approval of the study protocol must also be provided in the manuscript.

Human research participants

Policy information about studies involving human research participants

Population characteristics	See above
Recruitment	Samples were obtained with informed consent and all protocols were approved by The Second Xiangya Hospital of Central South University Ethics Review Board (Scientific and Research Ethics Committee, S-02/2000). Written informed consent was obtained from all patients. Written informed consent was obtained from the next of kin, caretakers, or guardians on the behalf of the minors/children participants involved in this study. Clinical samples from platinum based neoadjuvant chemotherapy resistant NSCLC patients were obtained from the Third Affiliated Hospital of Kunming Medical University and Harbin Medical University Cancer Hospital in China.
Ethics oversight	Samples were obtained with informed consent and all protocols were approved by The Second Xiangya Hospital of Central South University Ethics Review Board (Scientific and Research Ethics Committee, S-02/2000). Written informed consent was obtained from all patients. Written informed consent was obtained from the next of kin, caretakers, or guardians on the behalf of the minors/children participants involved in this study. Clinical samples from platinum based neoadjuvant chemotherapy resistant NSCLC patients were obtained from the Third Affiliated Hospital of Kunming Medical University and Harbin Medical University Cancer Hospital in China.

Note that full information on the approval of the study protocol must also be provided in the manuscript.

Clinical data

Policy information about clinical studies

All manuscripts should comply with the ICMJE guidelines for publication of clinical research and a completed CONSORT checklist must be included with all submissions.

Clinical trial registration	n/a
Study protocol	n/a
Data collection	n/a
Outcomes	n/a

Flow Cytometry

Plots

Confirm that:

- [x] The axis labels state the marker and fluorochrome used (e.g. CD4-FITC).
- [x] The axis scales are clearly visible. Include numbers along axes only for bottom left plot of group (a 'group' is an analysis of identical markers).
- [x] All plots are contour plots with outliers or pseudocolor plots.
- [x] A numerical value for number of cells or percentage (with statistics) is provided.

Methodology

Sample preparation	Fresh tumor samples and counterpart normal lung tissues, or tumor biopsy specimens were collected. The mice were euthanized by cervical dislocation when they became moribund, lungs were isolated, and cancer tissues were harvested.
Instrument	Polysome profiling was analyzed by NanoDrop (Thermo Fisher Scientific) for OD260.

Software	cBioPartal database query was used to analyze YTHDF1 expression in solid tumors.
Cell population abundance	n/a
Gating strategy	Cells were gated based on forward and side scatter plots, only avoiding debris and aggregates and no extensive gating strategy.

☒ Tick this box to confirm that a figure exemplifying the gating strategy is provided in the Supplementary Information.
